# Approaches to mapping genetically correlated complex traits

**DOI:** 10.1186/1471-2156-4-S1-S71

**Published:** 2003-12-31

**Authors:** Andrew W George, Saonli Basu, Na Li, Joseph H Rothstein, Solveig K Sieberts, William Stewart, Ellen M Wijsman, Elizabeth A Thompson

**Affiliations:** 1 Department of Statistics, University of Washington, Seattle, USA; 2 Department of Biostatistics, University of Washington, Seattle, USA; 3 Division of Medical Genetics, Department of Medicine, University of Washington, Seattle, USA

## Abstract

Our Markov chain Monte Carlo (MCMC) methods were used in linkage analyses of the Framingham Heart Study data using all available pedigrees. Our goal was to detect and map loci associated with covariate-adjusted traits log triglyceride (lnTG) and high-density lipoprotein cholesterol (HDL) using multipoint LOD score analysis, Bayesian oligogenic linkage analysis and identity-by-descent (IBD) scoring methods. Each method used all marker data for all markers on a chromosome. Bayesian linkage analysis detected a linkage signal on chromosome 7 for lnTG and HDL, corroborating previously published results. However, these results were not replicated in a classical linkage analysis of the data or by using IBD scoring methods.

We conclude that Bayesian linkage analysis provides a powerful paradigm for mapping trait loci but interpretation of the Bayesian linkage signals is subjective. In the absence of a LOD score method accommodating genetically complex traits and linkage heterogeneity, validation of these signals remains elusive.

## Background

The aim of our analyses was to detect and localize trait loci associated with quantitative traits logtriglyceride (lnTG) and high-density lipoprotein cholesterol (HDL) in the Framingham Heart Study data. Based primarily on Shearman et al. [1], we focused our search on certain chromosomes. By using Markov chain Monte Carlo analysis (MCMC), we were able to perform linkage analyses of data collected on large and sometimes complex pedigrees, using information from many marker loci simultaneously. Here we report multipoint linkage results from several approaches to analyzing the data.

## Methods

### Pedigree and map analysis

We first did routine analysis and investigation, removing uninformative individuals. Based on data availability, we decided for later analyses to use time point 11 for Cohort 1 and time point 1 for Cohort 2. For a few covariates unavailable at time point 11 for Cohort 1, the information was taken from time point 10 or 12. The program ECLIPSE was used to investigate pedigree uncertainties or errors on the basis of all available marker data. We also estimated sex-averaged and sex-specific recombination frequencies via an expectation maximization (EM) algorithm based on the MCMC program lm_auto in the MORGAN package (URL information below).

### Trait definition and segregation analyses

We analyzed quantitative traits using the MORGAN EM program PolyEM for fitting multivariate polygenic models. We used the phenotypic traits and covariates shown in Table 1. As a result of these analyses, we focused on HDL and lnTG for further study, defining the traits HDLA, lnTGA, and HDLAA as shown in Table 1.

Two approaches were used to obtain models for HDLAA to be used in linkage analysis. Loki [2], the Bayesian MCMC program for oligogenic models, provided some initial models. Also, normal mixtures were fitted to the adjusted trait values in a commingling analysis assuming Hardy-Weinberg equilibrium. Several binary traits were defined, with cutoffs at -10, +10, and +27.6 for both HDLAA and HDLA, this corresponding to 21% (23%), 19% (20%), and 3% (3%) of the observed individuals having the low, high, or very high HDLAA (HDLA) phenotype. An ordinal trait with 15 ordered categories was also used. Penetrances were defined for the binary trait and ordinal traits: for HDLAA the model was based on the commingling analysis and for HDLA on Loki output.

### Linkage detection and mapping

Our linkage studies focused primarily on chromosome 7. Linkage signals have been reported on chromosome 7 for lnTG, HDL, and log(HDL/TG) [1,2], which we hoped to replicate. Additional analyses were carried out on chromosomes 3, 4, 9, 11, 16, and 20, either to attempt replication of reported signals or as a negative control. Except where indicated, our results and discussion refer only to chromosome 7.

We used Loki to analyze several quantitative traits based on HDL and lnTG. The binary HDLA and HDLAA traits were subjected to IBD scoring linkage detection methods based on lm_auto [3]. We used the MORGAN multipoint LOD score program SCHNELL [4] on the quantitative HDLAA trait. A few single-marker LOD scores for the quantitative HDLA trait at chromosome 7 markers were checked using FASTLINK [5]. Both the binary and ordinal HDLA and HDLAA were also analyzed with the MORGAN MCMC program lm_bayes, a pseudo-Bayesian approach to the estimation of multipoint LOD scores [6]. All reported analyses used the Haldane genetic map distances, and all multipoint analyses used all markers on a chromosome simultaneously. Except where stated, we used sex-averaged maps. All pedigrees were used unbroken.

## Results

### Pedigree and map analysis

An ECLIPSE analysis of putative sib trios identified individual 590513 in pedigree 27096 as being an unlikely member of the stated sibship. Whereas true sib trios give log-likelihood differences in the range 40 to 80 relative to half-sib alternatives, trios including this individual gave values close to 0. This pedigree has substantial missing marker data, suggesting there may have been Mendelian errors. The data set that was left after removing 35 individuals with no data, 593 unobserved founder couples each with only one offspring, and the individual 590513, consisted of 3470 individuals in 362 pedigree components, ranging in size from 1 to 74. Two pedigrees contained loops due to sibship exchanges, and several had more than one founder couple. Our segregation and joint linkage/segregation analyses used the reduced data set of 3470 potentially informative individuals. Genome sharing, map re-estimation, and LOD score methods used the subset of 3444 individuals in non-singleton pedigrees.

Re-estimated maximum likelihood recombination frequencies were obtained for chromosome 7. The genetic distance from marker 7_1 to marker 7_22 increased from 191 cM (given) to 205 cM (estimated). Overall, there were few large differences between the given and estimated sex-averaged and sex-specific maps. Marker intervals 7_13–14, 7_18–19 and 7_19–20 showed the highest relative increase in sex-averaged recombination rates: 37%, 45%, and 58%, respectively.

### Trait definition and segregation analyses

The results of two polygenic segregation analyses are shown in Table 2. Joint analysis of HDLA and lnTGA showed substantial narrow-sense heritability for each trait (45% and 46%, respectively) and large negative genetic and environmental correlations. Adjusting HDLA for lnTG to define the HDLAA trait had little impact on the HDLA model parameters. Relative to an environmental model, the log-likelihood increases for a model including additive genetic effects were 141 and 73, under the bivariate and univariate analyses, respectively.

### Linkage detection and mapping

**Figure 1:**
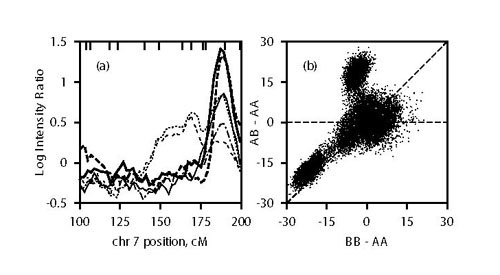
**Results from Loki **Chromosome 7 results from Loki. (a) Log10 IR for HDL (heavy dashed); HDLA (heavy solid); HDLAA (light solid); log(TG/HDL) with covariates CV1 and CV2 (dotted); HDLA with covariates CV2 and lnTG (dash-dot); and lnTG with CV1 and CV2 (double dashed). (b) Difference in genotype values for the heterozygote vs. AA homozygote (AB-AA) and BB homozygote vs. AA homozygote (BB-AA) for all iterations with QTL located between 175 and 195 cM, for a run based on HDLA (Table 1). Dashed lines indicate positions of dominant models. Positions of markers 11–22 are indicated along the top.

Bayesian analyses using Loki detected one main signal on chromosome 7 around marker 7_21. Figure 1a shows the log-intensity ratios (logIR) [7] for various traits. HDL and HDLA gave the strongest signals. The signal is weaker when HDL is adjusted for lnTG, suggesting that this signal is, at least in part, a TG linkage signal. lnTG and log(TG/HDL) showed weaker signals centered around marker 7_17. Additional positive signals were observed at 3_18–19 for HDLAA (logIR = 0.5), at 16_7 for HDLA with covariates CV2 and lnTG (logIR = 0.3) and 20_6 for lnTG with covariates CV1 and CV2 (logIR = 1.3).

The strength of Loki signals was sensitive to the prior distribution assumed for QTL effects. It was also sensitive to changes in the marker genetic map: the MCMC EM estimated map resulted in reductions in the peak IR, relative to the supplied map, of 11.6% and 17.3% for HDLA and lnTGA, respectively. As shown in Figure 1b, the realized QTL in the region 7_19 to 7_22 having non-negligible trait contributions were used to help define trait models for HDLA and HDLAA LOD score analyses.

**Figure 2:**
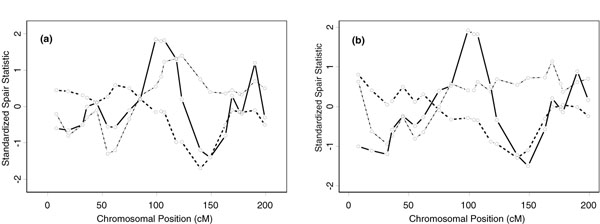
**S-pairs standardized statistics at marker locus positions on chromosome 7 **S-pairs standardized statistics at marker locus positions on chromosome 7: (a) HDLAA, (b) HDLA. Solid line: very high (>27.6). Semi-dashed line: low (≤10). Dashed line: high (>10).

Using the IBD S-pairs scoring statistic of [8], lm_auto gave weak signs of linkage in the region 7_11 and 7_21 for very-high HDLAA trait, with weaker signals for low HDLAA (Figure 2a). For HDLA, the signal in the region 7_21 was reduced (Figure 2b). Very high HDLAA also gave a signal at 3_18, consistent with the Loki signal.

**Figure 3:**
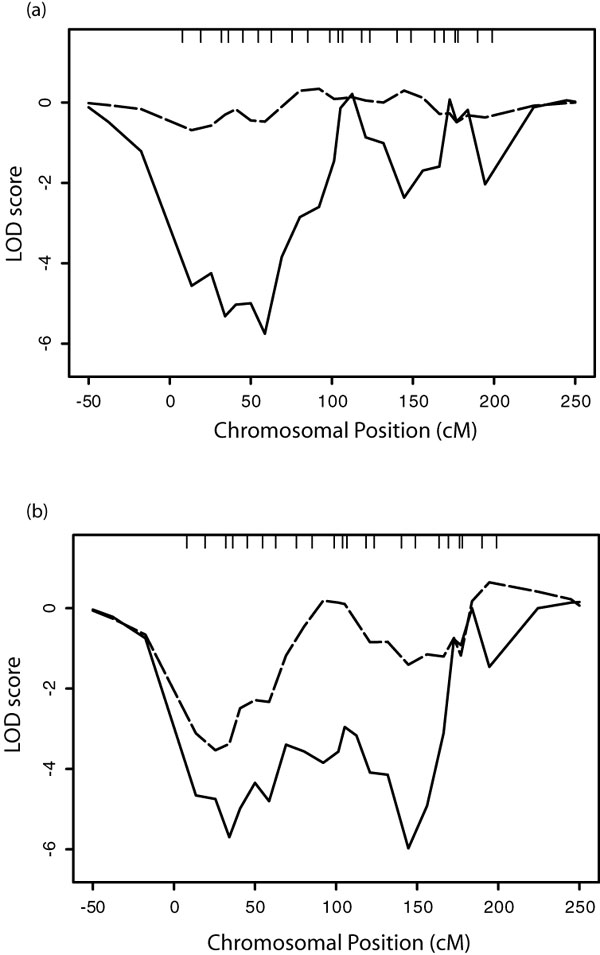
**Multipoint LOD scores on chromosome 7 **LOD scores on chromosome 7 for the very-high (>27.6) binary trait and the 15-category ordinal trait for (a) HDLAA and (b) HDLA. Dashed line, binary trait; solid line, ordinal trait.

Using penetrances based on commingling and Loki analyses, LOD scores for the "very-high" binary and ordinal HDLA and HDLAA traits were computed using lm_bayes (Figure 3). Although estimated LOD scores were barely positive, the curves do show the greater discriminating power provided by an ordinal compared to binary trait, and by not adjusting HDLA for lnTGA. SCHNELL also failed to obtain positive multipoint LOD scores for the quantitative HDLAA trait. No multipoint LOD score program found consistent positive signals for linkage, and there was sensitivity of LOD scores both to genetic map and model parameters. For example, analyzing the binary HDLAA trait under a sex-averaged map as apposed to a sex-specific map resulted in more negative multipoint LOD scores, with a drop of 2 LOD score units in some places. Interestingly, lm_bayes for the ordinal HDLA trait and FASTLINK for the quantitative HDLA trait gave single-marker LOD scores just above 1.0 close to marker 7_21.

## Conclusions

In combination, our segregation and linkage analysis results suggest both oligogenic inheritance of HDLA and lnTGA, and a negative genetic correlation, which may be the result of loci affecting these traits at chromosome 7-qter. The chromosome 7 signals were consistently stronger for HDLA than for HDLAA, the latter trait being adjusted for lnTG. For genetically correlated traits, adjustment may weaken the signal, whereas the ratio-trait of Shearman et al. [1] will reinforce the signal in the presence of a negative genetic correlation. Adjustments for genetically correlated covariates should be applied cautiously.

The weak signals of the model-free analyses of binary traits may be due to low power, and the problems of the multipoint LOD score analyses due to model sensitivity. In the presence of oligogenic inheritance, Loki can detect weak signals, imputing linked QTL only in certain families and modeling other heritable variation with unlinked QTL. However, interpretation of the strength of the signal provided by Loki remains an open question. Thus, in the absence of a LOD score method accommodating genetically complex traits and linkage heterogeneity, validation of these signals remains elusive.

## Figures and Tables

**Table 1:** Definition of phenotypes used in reported analyses

**Phenotype Notation**	**Definition or Computation**	**Trait (T) and/or Covariate (C)**
HDL	Fasting HDL cholesterol (mg/dl)	T
TG	Fasting triglycerides (mg/dl)	T
lnTG	Log_e_(TG)	T/C
BMI	707 × Weight/(height^2^)	C
Smk	Number of cigarettes smoked/day	C
Drk	Number of grams of alcohol/day	C
Gl	Fasting glucose (mg/dl)	C
CV1	Covariate set 1: age, sex, cohort	C
CV2	Covariate set 2: Drk, BMI	C
HDLA & lnTGA	HDL and lnTG adjusted for CV1	T
HDLAA	HDLA adjusted for CV2 and lnTG	T

**Table 2:** PolyEM Analysis of HDLA and lnTGA^A^

		**Bivariate Analysis**		**Univariate Analysis of HDLA**
			
		**HDLA**	**lnTGA**	
Overall Mean		0.242	-0.011	0.15
Variance	Additive Genetic	71	0.13	68
		88	0.15	90
Correlation	Additive Genetic	-0.46		
	Residual	-0.23		
Covariates used in trait preadjustment		CV1		CV1
Covariates used within trait analysis		BMI, Smk, Drk, Gl		Drk, CV2

^A^Bivariate PolyEM Analysis for HDLA and lnTGA, and analysis of HDLA. Residuals in the latter analysis define the HDLAA trait. Covariate categories are defined as follows: BMI (<18.5, 18.5–25, 25–30, >30); Smk (<1, 1–10, 11–20, >20); Drk (<1, 1–6, 7–18, >18); Gl (<82, 82–94, 95–101, >101); lnTG (< ln(200), > ln(200)).
